# Highly different risk estimates for atypical femoral fracture with use of bisphosphonates – debate must be allowed!

**DOI:** 10.3109/17453674.2012.718517

**Published:** 2012-08-25

Recently, 2 studies, published in prestigious journals, have presented widely different relative risk values for atypical femoral fractures with the use of bisphosphonates: 47 by Schilcher et al. (2011) and 2 by Feldstein et al. (2012). These surprisingly different figures merit an open discussion between authors. Accordingly, Schilcher et al. wrote a Letter to the Journal of Bone and Mineral Research, in which the Feldstein study was published, suggesting some explanations for the different results. However, JBMR rejected the letter and instead published an Editorial in support of the Feldstein study. The authors then sent the Letter to Acta Orthopaedica and requested publication. Acta sent the Letter to Feldstein and coauthors but they chose not to respond.

I think these remarkably disparate results merit a public discussion and Acta Orthopaedica is therefore publishing the Letter by Schilcher and colleagues that was originally sent to JBMR.
