# Supplementary material for: Highly different risk estimates for atypical femoral fracture with use of bisphosphonates – debate must be allowed!
Source: Acta Orthop. 2012 Aug 25;83(4):319–20. doi: 10.3109/17453674.2012.718517 (PMC3427619; doi:10.3109/17453674.2012.718517)
Supplement: Related texts

# The Letter sent to JBMR

**Published:** 2012-08-25

**Authors:** Per Aspenberg, Jörg Schilcher, Karl Michaëlsson

## To the Editor of JBMR

We would like to comment on the recent paper by Feldstein et al. on atypical fractures of the femur (1). These fractures were first reported by orthopedic surgeons who were astounded to see typical fatigue type fractures in a non-athletic elderly population. The fracture pattern, also typical for fatigue in many other materials, has been known for over a century, and when this pattern appeared in elderly women, the possible association between material fatigue and bisphosphonate use was conspicuous. Since then, the field has become confused by the introduction of the term “atypical” fracture, which was first used in epidemiologic studies meaning a subtrochanteric location. The new term was then redefined by the ASBMR task force, which introduced criteria for atypical fracture that somewhat vaguely describe a fatigue-type fracture (2). The main feature of this subtype of femoral shaft and subtrochanteric fractures, which struck those who first observed them, is that the fracture line on the lateral side is straight and virtually perpendicular to the cortex.

When this feature is included in the definition, the association between atypical fractures and bisphosphonate use is strong. A Swedish nation-wide, not industry-funded study, in which radiographs from 97% of all shaft or subtrochanteric fractures were reviewed, found a relative risk of 47 for atypical fractures with use of bisphosphonates identified from a complete prescription register. On average, the increase in absolute risk was 5 per 10,000 patient years (3). The recent paper by Feldstein et al. on atypical fractures – financed and partly performed by Merck – reports a relative risk of only 2, which is hard to reconcile with the results of the Swedish study by Schilcher et al.

There are some clues that could explain part of the discrepancy. Feldstein et al. claim that their cohort is similar to the local population, but the support in the manuscript for this claim is meager, as it is mainly based on a reference to a randomized trial in osteoporotic women. The results were only partly based on radiographic review, as 25% of the cases included in the analysis had no radiographs available. Shaft fractures were widely defined, as also fractures engaging the trochanteric region were included. Still, more than a third of these widely defined shaft fractures were classified as atypical The proportion showing the “ASBMR minor criteria” (a more sharply defined group) in Feldstein's study was only 29% of all claimed atypical fractures, whereas Schilcher et al had 80%. The Schilcher study identified 59 atypical fracture cases among 12,777 women with incident femoral fracture, whereas Feldstein et al. categorized 75 fractures as atypical out of less than 4000 incident femoral fracture cases. In addition, the ratio of atypical fractures to radiologically classified femoral shaft fractures was 0.62 in Feldstein's study whereas the same ratio was 0.22 in Schilcher's study. All together, these facts strongly indicate that Feldstein et al. used wide or unclear criteria for their definition of an atypical fracture. In consequence, the reviewers of the radiographs in Feldstein's study had only a moderate agreement (kappa 0.6) whereas the two blinded examiners in Schilcher's study had complete agreement.

Again, the fracture line perpendicular to the lateral cortex, typical of material fatigue, is easy to identify. The image provided by Feldstein et al. as an example of an atypical fracture does not display this fracture pattern, which has been shown in illustrations of numerous case reports, and which originally caused orthopedic surgeons to sound the alarm about fatigue fractures in the elderly. The published x-ray also shows a fracture line extending into the greater trochanter. This excludes classification as an atypical fracture and is a typical feature of a fragility type fracture. Because the number of atypical fractures is blown up by including fractures like the one in the published x-ray, the relative risk associated with bisphosphonate use becomes attenuated.

Moreover, there is a suggestion about glucocorticoids as a risk factor of atypical fractures in the abstract, whereas in fact a smaller proportion of the atypical fracture patients had been taking these drugs. If the authors had succeeded in identifying cases with the typical fatigue fracture pattern, an increased prevalence of glucocorticoid use ought to be expected since prolonged glucocorticoid treatment is a common indication for bisphosphonate use. Only bisphosphonates, and no other risk related drugs, were associated with atypical fractures in Schilcher's study.

Feldstein et al. finish their abstract claiming that there is a multitude of associated risk factors for atypical fractures. This statement is not supported by their data. Because of methodological shortcomings, especially regarding fracture classification, they were unable to show the strong relation between bisphosphonate use and atypical (fatigue-type) fractures, which was found in the so far only study covering an entire nation with radiographic adjudication. In fact, the association between bisphosphonate use and these fractures (3) was stronger than the one between tobacco smoking and lung cancer (4), a relation doubted for a long time by some researchers.

**Figure:** Radiograph from the paper by Feldstein et al. (2012) of a fracture classified as “atypical”. There is a fracture line extending into the greater trochanter (arrows inserted by Acta) which is a typical feature of a fragility type fracture and excludes classification as an atypical fracture. (Published with permission from JBMR).

## References

[CIT0001] Feldstein A, Black D, Perrin N, Rosales AG, Friess D, Boardman D, Dell R, Santora A, Chandler JM, Rix MM, Orwoll E (2012). Incidence and demography of femur fractures with and without atypical features. J Bone Miner Res.

[CIT0002] Shane E, Burr D, Ebeling PR, Abrahamsen B, Adler RA, Brown TD, Cheung AM, Cosman F, Curtis JR, Dell R, Dempster D, Einhorn TA, Genant HK, Geusens P, Klaushofer K, Koval K, Lane JM, McKiernan F, McKinney R, Ng A, Nieves J, O’Keefe R, Papapoulos S, Sen HT, van der Meulen MC, Weinstein RS, Whyte M (2010). Atypical subtrochanteric and diaphyseal femoral fractures: report of a task force of the American Society for Bone and Mineral Research. J Bone Miner Res.

[CIT0003] Schilcher J, Michaelsson K, Aspenberg P (2011). Bisphosphonate use and atypical fractures of the femoral shaft. N Engl J Med.

[CIT0004] Alberg AJ, Ford JG, Samet JM (2007). Epidemiology of lung cancer: ACCP evidence-based clinical practice guidelines (2nd edition). Chest.
